# Unexpected delivery: a case report of cryptic pregnancy in Nigeria

**DOI:** 10.11604/pamj.2020.36.205.23790

**Published:** 2020-07-22

**Authors:** Uloaku Akubueze Nto-Ezimah, Nto Johnson Nto, Emmanuel Anayochukwu Esom, Chika Juliet Okwor, Charles Adiri

**Affiliations:** 1 Department of Chemical Pathology, Faculty of Medical Sciences, College of Medicine, University of Nigeria, Enugu Campus, Enugu, Nsukka, Nigeria,; 2 Department of Anatomy, Faculty of Basic Medical Sciences, College of Medicine, University of Nigeria, Enugu Campus, Enugu, Nsukka, Nigeria,; 3 Department of Obstetrics and Gynecology, Faculty of Medical Sciences, College of Medicine, University of Nigeria, Nsukka, Nigeria

**Keywords:** Cryptic pregnancy, denial of pregnancy, neonatal risk, delivery, gestation, Nigeria

## Abstract

The etiology of cryptic pregnancy has not been fully elucidated and there exist misconceptions about this phenomenon in our contemporary Nigerian society. This case presents the first case report of cryptic pregnancy in sub-Saharan Africa. A case of a 19 year old overweight female student who presented to the sick bay at 01: 30 hours with a 3-day history of lower back pain, abdominal discomfort and constipation. At the sick bay the general practitioner on call asked if she was pregnant and she vehemently said no, recounting that she sees her menstruation regularly. Abdominal examination however, revealed a gravid uterus of about 36 weeks and vaginal examination showed a fully dilated cervix. She was surprised, terrified and confused and only remembered having unprotected sexual intercourse many months earlier. Barely two hours later, she gave birth via spontaneous vaginal delivery, to a live female infant at 03: 14 hours. This case emphasizes the need for general practitioners and specialists in sub-Saharan Africa to be aware of the phenomenon of cryptic pregnancy, which involves women not being conscious of their gravid state until final weeks of gestation or at delivery, to reduce neonatal and maternal complications.

## Introduction

Pregnancy also known as gestation involves a sequence of events which start from fertilization of the ovum by a spermatozoon, implantation, embryogenesis to embryonic and fetal development. It lasts for 38-40 weeks. The symptoms of pregnancy include nausea, vomiting, raised basal temperature, amenorrhea, breast and abdominal enlargement. Fetal movement may be felt at 18-20 weeks for primigravidae and 16 weeks for multigravidae. Some women in their gravid state may have pseudomenstrual bleeding and mild or absent pregnancy related symptoms [1-5]. They may have mild labor and pain during delivery [2-5]. Cryptic pregnancy or denial of pregnancy is defined as lack of subjective awareness of pregnancy until final weeks of gestation or delivery [1,4,5]. Their relatives, family doctors and sexual partners are also not aware of their gravid state [1,2,6]. Cryptic pregnancy differs from concealed pregnancy; in the latter the women are aware of their gravid state but deliberately keep it secret [1,4,5]. Cryptic pregnancy has been classified into three subtypes: affective, psychotic and pervasive [7]. The etiology of this fascinating phenomenon has not been fully elucidated [5]. Del Guidice explained cryptic pregnancy from a parent-offspring conflict theory, where the mother benefits at the expense of the child, [2] while Sandox expounded: *“a stand by-in-tension response to an unresolved intrapersonal conflict opposing pro- and against-pregnancy forces”* [5]. Wessel *et al*. [8] opined that women who experienced pregnancy denial were heterogeneous and there are no clear cut characteristics of a person who experiences pregnancy denial, however, this has been reported in mostly young, unmarried and immature women [1,6]. Cryptic pregnancy was earlier assumed to be rare and associated with mothers´ mental health [2,3,5,9]. However, it has been revealed that the phenomenon is more prevalent than earlier perceived [2,6]. In Germany the prevalence of cryptic pregnancy discovered after 20 weeks of gestation is 1: 475 and 1: 2500 for unanticipated deliveries [2,6,8,10]. It is estimated at 1: 400 deliveries in Australia [6,11] and 1: 500 deliveries in the United States [6]. There is dearth of data in scientific literature and a misconception on cryptic pregnancy in sub-Saharan Africa. This article presents the first case report on cryptic pregnancy in Nigeria.

## Patient and observation

A 19 year old overweight female student, Ms K, in Enugu State, South East, Nigeria, who presented to a sick bay at 01: 30 hours with a 3 day history of lower back pain, abdominal discomfort and constipation. Unknown to her, she was in labor. She had reported to the outpatient department of a hospital several months ago with complains of malaise repeatedly overtime; her general practitioner (GP) treated empirically for malaria on each visit with same complaints and the recount that her menstruation was regular. At the sick bay the GP on call asked if she was pregnant and she vehemently said no, recounting that she sees her menstruation regularly. Abdominal examination however, revealed a gravid uterus of about 36 weeks and vaginal examination showed that her cervix was fully dilated. When confronted with the situation, Ms K, was surprised as well as confused. However, she only remembered having unprotected sexual intercourse many months ago. The baby presented in a frank breech position. To facilitate delivery a Pinard maneuver was done. She gave birth via spontaneous vaginal delivery to a live female baby weighing 3 kg at 03: 14 hours. Labor and delivery pain was minimal. Ms K was in absolute shock, she refused to touch the baby, it was dramatic as she struggled to accept that she just became a mother. As a result of the circumstances of the delivery, altered judgement of the mother and the potential neonatal risk; the GP insisted and invited the parents. Her parents arrived at 8: 30 hours, upon sighting the baby they inquired whose it was. They were completely baffled to know the baby was their granddaughter. It was even more difficult for the sudden grandmother, a nurse, who kept wondering how her daughter´s gravid state eluded her. Due to the peculiarities of the delivery the GP at the sick bay followed up the case, the baby was christened Peace. The GP asked why the name Peace. Ms K replied *“the birth of the baby brought unresolved conflict between her family and the male partner´s family”*. It was after the baby was born that she realized the reason behind all that she had gone through. She had nausea only when she drank cold water, this later stopped. She admitted noticing slight increase in weight and mild abdominal protrusion, this made her do exercises which didn´t help. She believes her overweight made her and her relatives not to suspect pregnancy. Ms K was confused as she just couldn´t understand what was going on with her body and her GP, who always treated malaria without proper investigation, was of no help either. Ms K said she would have committed suicide if her gravid state was discovered before delivery. When asked why she replied *“how on earth was she going to explain her gravid state to her parents”*.

## Discussion

Pregnancy is one of life´s most celebrated events. However, overwhelming doubts and fears have made some women imbibe unconscious defence mechanisms to refuse the basic reality of their gravid state. This case report revealed genuine surprise for Ms K and her parents and suggests she was unaware of her pregnancy. Ms K recalled having nausea at some point and noticed weight gain and mild abdominal protrusion but couldn´t have thought of pregnancy being that her menstruation was regular. It has been documented that women who experience denial of pregnancy perceive their gravid state but are unable to relate these symptoms to pregnancy [6]. The apparent paradox in the denial of pregnancy is the report of menstruation-like bleeding [6,11,12]. It has been observed that irregular and continuous non menstrual bleeding has made some women misinterpret or deny their own pregnancy [6,11]. Wessel and Endrikat [12] evaluated pseudomenstruation in women who experience regular bleeding in cryptic pregnancy and elucidated that postnatal hormonal patterns couldn´t explain the cyclic menstruation-like bleeding reported during denied pregnancies.

Relatives, family doctors and sexual partners are of paramount importance in such situation, nonetheless they also are not aware of the gravid state [1,2,6]. Melanin Klein, an Australian-British psychoanalyst (referenced by Goncalves [6]) explained why family doctors do not detect pregnancy in these women; she noted that the women project their unconscious wish of not been pregnant towards the doctors; the doctors become enchanted by this attribution thereby making diagnostic errors by not examining properly. Wessel *et al*. [13] agreed with the defence mechanism of projective identification and referred to the dynamics as iatrogenic participation [6,14].

This emphasizes the need for doctors to request for a pregnancy test in women of reproductive age who present with recurrent pregnancy-related symptoms irrespective of whether menstruation is regular or not. That would have been the case for Ms K in this present report. If the doctors had recalled the recurrent malaria like symptoms and examined her properly and meticulously as well request investigations such as pregnancy test, it would have averted the dangers of the bad fate that Ms K and her baby would have faced if the GP at the point of labour did not do a painstaking examination as to confirm both the gravid state as well as the advanced labour signs.

Denial of pregnancy puts the mother and foetus at significant risks as result of lack of antenatal care, poor nutrition, fetal abuse, unsupervised and or precipitate delivery [1,4,6]. Ms K had no prenatal care, was not exposed to healthy pregnancy diet and attempted weight lose via exercise during her pregnancy. Most of the documented cases of pregnancy denial turn out just fine. Nonetheless, under certain circumstances the result could be tragic. Ms K´s baby presented breech. Brezinka *et al*. [11] evaluated the obstetrical aspects of pregnancy denial and reported that in 11 cases of cryptic pregnancy discovered at delivery, five of them had breech presentation. Vaginal birth by breech possess significant risk and this happened at an odd hour at a sick bay in a poor resource setting. Awareness of the complications of pregnancy denial would be valuable in reducing maternal and infant mortality.

Goncalves, [6] opined *“after finding out about an unknown pregnancy, mothers may face some struggle with their new born”* Ms K found it difficult to accept her baby. Infanticide is the most dangerous consequence of pregnancy denial and may not be premeditated [6].

As a result of altered judgement the mother may not be aware of what she is doing. It is important that health care providers recognized this and ensure the safety of the baby and the mother. The birth weight was normal and similar to the birth weights in the cases reported by Neifert and Bourgeois [1], Stammers and Long [4] and Goncalves [6].

In Southern Nigeria there is an illusion about cryptic pregnancy, a big money spinning scam, as reported in an article captioned *“inside Imo´s wonder pregnancy centre”* in one of Nigerian dailies, The Nation, published on the 5^th^of October, 2019. Childless couples are made to believe that pregnancy can be created. The unsuspecting couple are made to pay huge sums, the women put through unethical and non-medical procedures and are strictly warned not to go for antenatal visits at a hospital. The syndicates claim the child is hidden and medical diagnostics may not detect the pregnancy. The women continue to have their monthly menstrual flow during the pregnancy and can only be delivered of their baby at the centre. The women are made to believe they are pregnant and in the end they are given babies taken from young teenage mothers. There is need for health care providers to correct this impression through awareness campaigns.

This case demonstrated how the diagnosis of cryptic pregnancy is difficult and why a high index of suspicion is vital in recognizing the condition especially in resource poor settings or countries. In this case the diagnosis was completely missed all through pregnancy and emphasize the need for doctors to mindful of the phenomenon.

## Conclusion

This case emphasizes the need for general practitioners and specialists in sub-Saharan Africa to be aware of the phenomenon of cryptic pregnancy, which involves women not being conscious of their gravid state until final weeks of gestation or at delivery, to reduce neonatal and maternal complications.
